# The temporal relationship between parental concern of overeating and childhood obesity considering genetic susceptibility: longitudinal results from the IDEFICS/I.Family study

**DOI:** 10.1186/s12966-021-01205-9

**Published:** 2021-11-04

**Authors:** Guiomar Masip, Ronja Foraita, Karri Silventoinen, Roger A. H. Adan, Wolfgang Ahrens, Stefaan De Henauw, Antje Hebestreit, Anna Keski-Rahkonen, Lauren Lissner, Kirsten Mehlig, Dénés Molnar, Luis A. Moreno, Iris Pigeot, Paola Russo, Toomas Veidebaum, Leonie H. Bogl, Jaakko Kaprio, on behalf of the IDEFICS/I.Family Consortia

**Affiliations:** 1 Department of Public Health, University of Helsinki, Helsinki, Finland; 2 Leibniz Institute for Prevention Research and Epidemiology – BIPS, Bremen, Germany; 3 Population Research Unit, Faculty of Social Sciences, University of Helsinki, Helsinki, Finland; 4 Department of Translational Neuroscience, UMC Utrecht Brain Center, University Medical Center Utrecht and Utrecht University, Utrecht, the Netherlands; 5 Faculty of Mathematics and Computer Science, University of Bremen, Bremen, Germany; 6 Department of Public Health and Primary Care, Faculty of Medicine and Health Sciences, Ghent University, Ghent, Belgium; 7 School of Public Health and Community Medicine, Institute of Medicine, Sahlgrenska Academy, University of Gothenburg, Gothenburg, Sweden; 8 Department of Paediatrics, Medical School, University of Pécs, Pécs, Hungary; 9 GENUD (Growth, Exercise, Nutrition and Development) Research Group, Faculty of Health Sciences, University of Zaragoza Instituto Agroalimentario de Aragón (IA2), Instituto de Investigación Sanitaria de Aragón, Zaragoza, Spain; 10 Centro de Investigación Biomédica en Red de Fisiopatología de la Obesidad y Nutrición CIBEROBN, Instituto de Salud Carlos III, Madrid, Spain; 11 Institute of Food Sciences, National Research Council, Avellino, Italy; 12 Department of Chronic Diseases, National Institute for Health Development, Tallinn, Estonia; 13 Department of Epidemiology, Center for Public Health, Medical University of Vienna, Vienna, Austria; 14 Institute for Molecular Medicine Finland (FIMM), University of Helsinki, Helsinki, Finland

**Keywords:** Obesity, Overeating, Polygenic risk score, Body mass index, Genetics, Temporal associations, Mediation

## Abstract

**Background:**

Many genes and molecular pathways are associated with obesity, but the mechanisms from genes to obesity are less well known. Eating behaviors represent a plausible pathway, but because the relationships of eating behaviors and obesity may be bi-directional, it remains challenging to resolve the underlying pathways. A longitudinal approach is needed to assess the contribution of genetic risk during the development of obesity in childhood. In this study we aim to examine the relationships between the polygenic risk score for body mass index (PRS-BMI), parental concern of overeating and obesity indices during childhood.

**Methods:**

The IDEFICS/I.Family study is a school-based multicenter pan-European cohort of children observed for 6 years (mean ± SD follow-up 5.8 ± 0.4). Children examined in 2007/2008 (wave 1) (mean ± SD age: 4.4 ± 1.1, range: 2–9 years), in 2009/2010 (wave 2) and in 2013/2014 (wave 3) were included. A total of 5112 children (49% girls) participated at waves 1, 2 and 3. For 2656 children with genome-wide data we constructed a PRS based on 2.1 million single nucleotide polymorphisms. Z-score BMI and z-score waist circumference (WC) were assessed and eating behaviors and relevant confounders were reported by parents via questionnaires. Parental concern of overeating was derived from principal component analyses from an eating behavior questionnaire.

**Results:**

In cross-lagged models, the prospective associations between z-score obesity indices and parental concern of overeating were bi-directional. In mediation models, the association between the PRS-BMI and parental concern of overeating at wave 3 was mediated by baseline z-BMI (*β* = 0.16, 95% CI: 0.10, 0.21) and baseline z-WC (*β* = 0.17, 95% CI: 0.11, 0.23). To a lesser extent, baseline parental concern of overeating also mediated the association between the PRS-BMI and z-BMI at wave 3 (*β* = 0.10, 95% CI: 0.07, 0.13) and z-WC at wave 3 (*β* = 0.09, 95% CI: 0.07, 0.12).

**Conclusions:**

The findings suggest that the prospective associations between obesity indices and parental concern of overeating are likely bi-directional, but obesity indices have a stronger association with future parental concern of overeating than vice versa. The findings suggest parental concern of overeating as a possible mediator in the genetic susceptibility to obesity and further highlight that other pathways are also involved. A better understanding of the genetic pathways that lead to childhood obesity can help to prevent weight gain.

**Trial registration:**

Registry number: ISRCTN62310987 Retrospectively registered 17 September 2018.

**Supplementary Information:**

The online version contains supplementary material available at 10.1186/s12966-021-01205-9.

## Introduction

Obesity is a heritable and highly polygenic chronic disease [1–4]. Genetic factors explain between 40 to 60% of the proportion of the variability in body mass index (BMI) during childhood [5]. With the advance of genomic research, there has been a shift from twin and family studies towards using measured genes in genome-wide association studies. To date, the largest meta-analysis of genome-wide association studies of BMI among adults has led to the discovery of 751 single nucleotide polymorphisms (SNPs) associated with BMI; these SNPs collectively explain 6% of the phenotypic variance in BMI [4]. Khera et al. (2019) estimated that 23% of the variation in BMI is accounted for by common variants. When rare variants are also assessed by whole genome sequencing, the proportion of variance in BMI explained by measured genetic variants rises to 40% [6], which is close to the heritability estimates from twin and family studies. Using information from 2.1 million measured and imputed common variants irrespective of genome-wide significance [7], the newest polygenic risk score (PRS) is a single measure that quantifies the inherited susceptibility to a disease [8].

Both genes and environment can influence behaviors and physiology and they are involved in the regulation of energy intake and energy expenditure [5]. A low basal metabolic rate is partly heritable (about 40%) [9, 10] and is a risk factor for weight gain [11]. Appetite traits are also partly heritable (50–85%) in infancy and childhood [12–14] and are related to weight gain [15]. However, more research is needed to identify what pathway is stronger, as it is not yet established whether obesity susceptibility genes are more likely to influence body weight through metabolism or appetite traits. Since obesity susceptibility genes are highly expressed in the central nervous system [3, 16, 17], most previous research has focused on the role of appetite traits as behavioral pathways to obesity [5]. Results from a British cross-sectional study of 10-year old twins [18] and from a prospective French study of 1 to 5-year old children [19] showed that appetite traits mediate the genetic susceptibility to childhood obesity.

These earlier studies have used PRS-BMI based on a small number of variants (32 or fewer) and thus had a lower predictive power for capturing the obesity genetic risk [7]. Three previous PRS-BMI mediation studies have included all available common genetic variants [20–22], but they did not examine the temporal direction of appetite traits and obesity. Longitudinal studies using genetically sensitive designs to investigate the direction of pathways between eating behaviors and weight gain are needed to advance our understanding in the field [23].

Thus, we examined the temporal relationship between parental concern of overeating and obesity indices to examine the pathways by which the genetic susceptibility to obesity expresses itself by using children’s longitudinal data from the IDEFICS/I.Family European multicenter cohort and the latest available PRS for BMI.

## Methods

### Participants

**Fig. 1:**
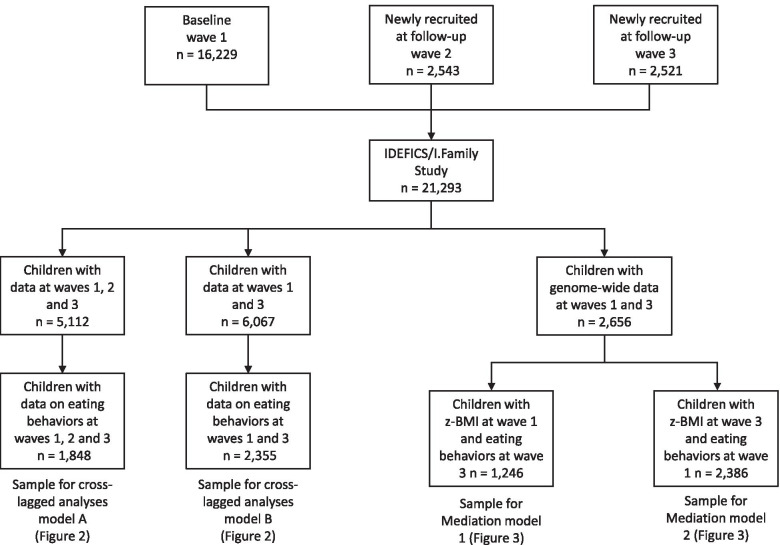
Flowchart diagram of participants

The IDEFICS/I.Family study is a pan-European multicenter cohort that aimed at investigating eating habits and lifestyle factors in children and adolescents from eight countries (Belgium, Cyprus, Estonia, Germany, Hungary, Italy, Spain, and Sweden) [24]. The baseline examination (wave 1) included 16,229 children aged 2 to 9.9 years old. Follow-up examinations took place 2 years (wave 2, *n* = 13,586) and 6 years later (wave 3, *n* = 9639) (Fig. 1). A total of 2656 children were genotyped. Children from Cyprus were not selected for genotyping to minimize population stratification. In waves 1 (baseline) and 2, parents (or legal guardians) completed a self-administered questionnaire about their children’s health and lifestyle. In wave 3, questionnaires were completed by parents or their legal guardians for children up to 11 years old and for themselves and also by children for themselves if aged ≥12 years. Children participating in the study were asked to donate venous or capillary blood and to provide a saliva sample for DNA [25].

Research ethics committees in each country approved the study in accordance with the ethical standards of the Declaration of Helsinki. All children and parents gave informed consent to participate in the study.

### Obesity indices

Height was measured with a portable stadiometer (Seca GmbH & Co. KG., Hamburg, Germany) to the nearest 0.1 cm and weight with a TANITA digital scale (TANITA Europe GmbH, Sindelfingen, Germany) to the nearest 0.1 kg. Both measurements were performed in the morning in fasting conditions and light clothing [26]. BMI was calculated from height and weight (kg/m^2^). Waist circumference was measured with an inelastic tape (Seca 200, Birmingham, UK) in the upright position with a relaxed abdomen at the midway point between the lowest rib and the iliac crest to the nearest 0.1 cm. Age- and sex- specific BMI z-scores (z-BMI) were calculated according to Cole [27] and age- and sex- specific WC z-scores (z-WC) were calculated according to the IDEFICS reference values [28].

### Family food environment

Information on the family food environment was collected by questionnaire at all study waves. Parents or legal guardians answered questions about eating- and weight-related behaviors as well as parental worries toward their child’s food style for children aged under 12 years. Children aged 12 or older were not administered this questionnaire.

Questions were selected from the questionnaire from Baughcum et al. [29], with 11-items at waves 1 and 2 and 10-items at wave 3. The questionnaire covered five constructs (difficulty on child feeding; concern about children overeating and becoming overweight; pushing the child to eat more; the situation and structure during feeding; age-inappropriate feeding), with five possible response categories from “never” to “always”. Parents answered two more items: “How concerned are you about your child eating too much when you are not around him/her?” with four possible responses from “unconcerned” to “concerned”, and “How often does your child eat doing something else (e.g. watching TV, playing, sitting at a computer, looking at a book)?” with four possible response categories from “never” to “on several occasions per day”.

### Derivation of eating behavior patterns

We derived eating behavior patterns by principal component analyses using 13-items (waves 1 and 2) and 12-items (wave 3) from the family food environment questionnaire described above and shown in Table S1 (Additional files). Based on eigenvalues > 0.80 we retained 4 components which explained 67% (wave 1) and 68% (waves 2 and 3) of the variance of the family food environment questions. Factor loadings ≥0.30 were considered to contribute to the eating behavior pattern and were used to label the 4 components. Out of them, parental concern of overeating was the component with the largest variance (about 30% in all three study waves) and included the following items: had to stop child from eating too much”; “thought about putting child on a diet to keep him/her from becoming overweight”; “worried about child eating too much” and “child eating too much when you are not around” (see Additional files, Table S1 for more details of the different eating behavior patterns).

### Genotyping and Quality Control & Polygenic scoring

DNA was extracted from either saliva or blood samples using standard methods. The samples of 3515 children were genotyped using the UK Biobank Axiom array (Thermo-Fisher Scientific, Santa Clara, USA) in two batches (2015 and 2017). Sample and genotype quality control measures were applied following the recommendations of Weale [30], genome-wide imputation was done with Minimac3 v5 (https://genome.sph.umich.edu/wiki/Minimac3) resulting in 3098 children and 3,424,677 genotypes with an estimated posterior genotype probability > 0.8 and a minor allele frequency ≥ 0.05. EMMAX (https://genome.sph.umich.edu/wiki/EMMAX) was applied to calculate a genetic relatedness matrix in order to estimate the degree of relatedness within the study sample. A PRS-BMI was calculated as proposed and validated by Khera et al. [7] using the same reference population from Locke et al. [3]. It consists of 2,100,302 SNPs and is based on genome-wide summary statistics for BMI from European ancestry populations (~ 300,000 samples) [3].

### Covariates

Six covariates (parental education, parental income, well-being, screen time duration, playing outside and fruit and vegetable consumption) were assessed by questionnaires. Glycated hemoglobin was analyzed by high-performance liquid chromatography.

Parents were asked to specify their education and income. Parental education was reported by one of the parents according to the International Standard Classification of Education (ISCED) [31], the maximum ISCED level of both parents was calculated. ISCED categories were as follows: low (levels 1–2); medium (levels 3–4) and high (levels 5–6). Parental income was asked according to the monthly net income of the household after taxes and deductions and categorized as follows: low, low-medium, medium, medium-high, and high. Parents filled out the KINDL® questionnaire on their child’s well-being and a score that ranges from 0 to 48 points was calculated [32].

Screen time in hours per week was calculated by summing up the total screen time spent on audiovisual media (TV, video, DVD, computer, game console) on weekdays and weekend days (5 x weekday + 2 x weekend).

Playing outdoors as a proxy for physical activity was calculated in hours per day and derived from hours playing outdoors on weekdays and hours playing outdoors on weekend days (5 x weekday + 2 x weekend)/7).

Information on fruits and vegetables consumption was calculated as the sum of the reported intake of raw and cooked vegetables and fresh fruit, which was obtained by a validated food frequency questionnaire [33, 34].

Glycated hemoglobin (HbA1c) was analyzed by high-performance liquid chromatography (AUTO-GA variant). Details on laboratory analyses can be found in Peplies et al. [35].

### Statistical analyses

Descriptive characteristics were presented as means and standard deviations (SDs) for continuous variables and as numbers and percentages for categorical variables. We used chained random forest imputation to replace missing data on covariates. Missing data were randomly imputed to increase the number of observations and to reduce the probability of bias that might result from excluding missing cases. Missing values were assumed to be missing at random and missing values were imputed using chaining random forests [36] as implemented in missRanger (https://CRAN.R-project.org/package=missRanger). The random forest imputation is based on 200 trees and was controlled for a variation of additional predictors. A huge advantage of using random forests is that they produce a single imputed dataset, they are adaptive to interactions and nonlinear relationships not needing to specify an imputation model, and they can handle mixed types of missing data.

**Fig. 2:**
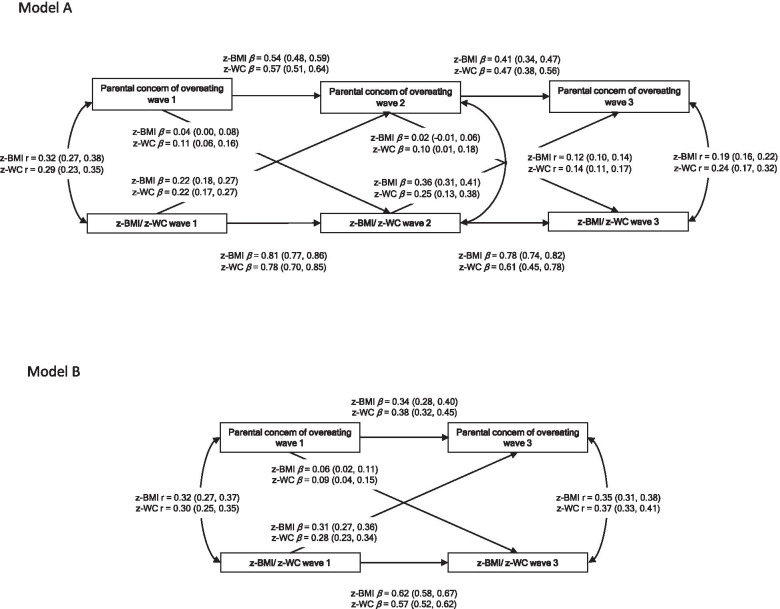
Cross-lagged path model for parental concern of overeating and obesity indices. Legend: Prospective associations between parental concern of overeating and z-BMI and z-WC. All models were adjusted for age, sex, country, parental education, parental income, well-being, screen time, playing outside, fruits and vegetables consumption and glycated hemoglobin. Model **A** *n* = 1848 for z-BMI; *n* = 1576 for z-WC; Model **B** *n* = 2355 for z-BMI; *n* = 2022 for z-WC. Abbreviations: z-score body mass index (z-BMI), z-score waist circumference (z-WC)

We conducted cross-lagged path models (CLPM) in a structural equation modeling framework to examine the temporal associations between parental concern of overeating and obesity indices (z-BMI and z-WC) (Fig. 2). Before CLPM, we regressed parental concern of overeating and obesity indices on age, sex, country, and the seven covariates listed above at each examination wave. We standardized the obtained residuals and used the standardized residuals in CLPM analyses. The effect of related siblings was taken into account by using survey methods with robust standard errors yielded by cluster variance estimators [37]. Goodness-of-fit criteria were a Comparative Fit Index (CFI), a Tucker-Lewis Index (TLI) ≥ 0.90, and a Root Mean Square Error of Approximation (RMSEA) close to 0.06 [38, 39].

**Fig. 3:**
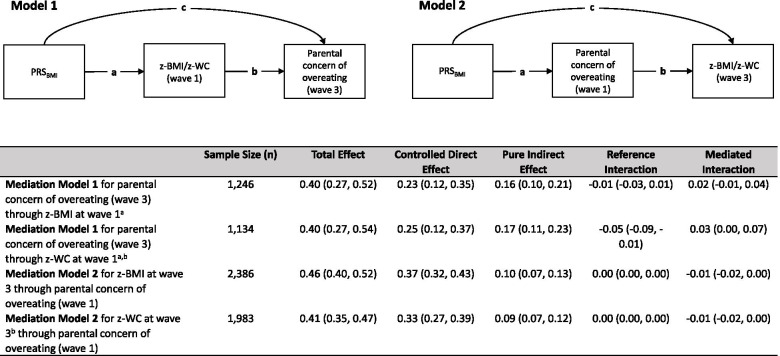
Results from the causal mediation models. Legend: Model 1: PRS-BMI (exposure), z-BMI and z-WC at wave 1 (mediators), parental concern of overeating at wave 3 (outcome). Model 2: PRS-BMI (exposure), parental concern of overeating at wave 1 (mediator), z-BMI and z-WC at wave 3 (outcomes). The indirect effect (or mediation effect) is represented by ab, and c represents the direct effect; total effect = c + ab. Results were presented as *β*-coefficients and their 95% CIs. All models were adjusted for baseline age, sex, country, parental education, parental income, well-being, screen time, playing outside, fruit and vegetable consumption, glycated hemoglobin and genetic principal components. ^a^Sample size smaller due to missing values on eating behaviors at wave 3. ^b^Sample size smaller due to missing values on z-WC. Abbreviations: polygenic risk score for body mass index (PRS-BMI), z-score body mass index (z-BMI), z-score waist circumference (z-WC)

To study whether obesity indices at baseline mediated the association between the PRS-BMI and parental concern of overeating at wave 3 (Fig. 3, Model 1) and whether parental concern of overeating at baseline mediated the association between the PRS-BMI and obesity indices at wave 3 (Fig. 3, Model 2), we conducted causal mediation analyses based on the counterfactual approach proposed by VanderWeele [40]. We used the med4way Stata package which allows for decomposing the total effect of an exposure to an outcome into four components: controlled direct effect, pure indirect effect (or mediation effect), reference interaction, and mediated interaction [41]. Sample bias correction estimates and 95% confidence intervals (CIs) were calculated using the bootstrapping approach of 1000 draws (Mediation Model 1) or 2000 draws (Mediation Model 2). We adjusted all models by baseline age, sex, country, the 7 covariates listed above and the first 12 genetic principal components. We a priori selected this set of confounders for which to control in the mediation models. We attempted to control for covariates that may be the cause of the exposure, or of the outcome, or of both [42]. The causal mediation analyses were further used to overcome the limitations of standard approaches, which do not account for mediator-outcome confounding. In case that there is mediator-outcome confounding, standard approaches are biased. For example, screen time, could be causally related to parental concern of overeating and to obesity indices, hence it is reasonable to include it as a confounder. We also conducted sensitivity analyses to examine the presence of unmeasured variables that may confound the relationship between the mediator and the outcome and to assess how strong are the assumptions about confounding that are needed to identify the direct and indirect effects [40].

### Description of the analysis dataset

For the cross-lagged analyses, we included all children with data on eating behaviors and obesity indices at waves 1, 2 and 3 (*n* = 1848 from 1689 families, see Fig. 2A). We also performed the same analyses including children only from waves 1 and 3 (*n* = 2355 from 2143 families, see Fig. 2B). In mediation analyses, we included all children with genome-wide data, obesity indices at wave 1 and eating behaviors at wave 3 (Mediation Model 1, *n* = 1246 children) and with genome-wide data, eating behaviors at wave 1 and obesity indices at wave 3 (Mediation Model 2, *n* = 2386 children). Figure 1 is a flowchart showing the derivation of the sample sizes.

## Results

### General characteristics

Table 1 summarizes the general characteristics of the children at waves 1 and 3. About half were girls and half of the children came from families with a high parental education level. Children’s z-BMI, z-WC, and screen time increased with age. Table S2 (Additional files) further shows the baseline characteristics of the children with available data on eating behaviors who participated in all three study waves.

**Table 1:** General characteristics of the study sample (*n* = 2355)

	Wave 1	Wave 3
Age in years, mean (SD)	4.4 (1.0)	10.1 (1.0)
Girls, n (%)	1124 (47.7)	1124 (47.7)
Country, n (%)		
Belgium	135 (5.7)	135 (5.7)
Cyprus	141 (6.0)	141 (6.0)
Estonia	388 (16.5)	388 (16.5)
Germany	370 (15.7)	370 (15.7)
Hungary	284 (12.1)	284 (12.1)
Italy	400 (17.0)	400 (17.0)
Spain	232 (9.9)	232 (9.9)
Sweden	405 (17.2)	405 (17.2)
z-score BMI, mean (SD)	−0.01 (1.1)	0.39 (1.2)
z-score WC, mean (SD)	−0.09 (1.2)	0.69 (1.2)
Missing	255	83
Parental education level, n (%)		
Low	104 (4.4)	97 (4.1)
Medium	979 (41.6)	979 (41.6)
High	1253 (53.2)	1279 (54.3)
Missing	19 (0.1)	–
Parental income level, n (%)		
Low	352 (14.9)	430 (18.3)
Low - Medium	399 (16.9)	147 (6.2)
Medium	620 (26.3)	903 (38.3)
Medium - High	375 (15.9)	270 (11.5)
High	487 (20.7)	605 (25.7)
Missing	122 (5.2)	–
Well-being score, mean (SD)	40.8 (4.2)	40.3 (4.5)
Missing	114	–
Screen time (hours/week), mean (SD)	9.7 (6.3)	14.4 (8.0)
Missing	52	–
Playing outside (hours/day), mean (SD)	2.3 (1.5)	1.7 (1.3)
Missing	248	506
Fruit and vegetable consumption (portions/week), mean (SD)	18.8 (11.0)	19.1 (11.5)
Missing	266	–
Glycated hemoglobin (%), mean (SD)	4.6 (0.5)	5.0 (0.3)
Missing	907	–

### Prospective associations between obesity indices and parental concern for overeating

Figure 2A shows the CLPMs of children’s parental concern of overeating and obesity indices considering all three available time points (waves 1, 2 and 3). The associations between z-WC to later parental concern of overeating were bi-directional, but the pathways from z-WC to later parental concern of overeating were stronger (*β* = 0.22, *p* < 0.001 from wave 1 to 2; *β* = 0.25, *p* < 0.001 from wave 2 to 3) than the reverse pathways (*β* = 0.11, *p* < 0.001 from waves 1 to 2; *β* = 0.10, *p* = 0.04 from waves 2 to 3). Similar associations from z-BMI to later parental concern of overeating were observed, but there were no significant associations in the opposite direction. The associations between z-BMI and z-WC to later parental concern of overeating were stronger than the other way around. The models had a reasonable fit to the data (RMSEA = 0.055, CFI = 0.996, TLI = 0.987 for z-BMI; and RMSEA = 0.078, CFI = 0.991, TLI = 0.967 for z-WC).

Figure 2B presents CLPMs of children’s parental concern of overeating and obesity indices at waves 1 and 3. We observed bi-directional associations between parental concern of overeating and both z-BMI and z-WC. The pathways from z-BMI and z-WC at wave 1 to later parental concern of overeating were stronger (*β* = 0.31, *p* < 0.001 and *β* = 0.28, p < 0.001, respectively) than the reverse pathways (*β* = 0.06, *p* = 0.006 for z-BMI; *β* = 0.09, p < 0.001 for z-WC). The models had a fit to the data (RMSEA = 0.000, CFI = 1.000, TLI = 1.000, for both models).

### Results from the causal mediation analyses

The results obtained from the causal mediation analyses (Fig. 3) showed that z-BMI and z-WC at wave 1 mediated the association between the PRS-BMI and parental concern of overeating at wave 3 (Fig. 3, Model 1). Parental concern of overeating at wave 1 also mediated the association between the PRS-BMI and both obesity indices at wave 3, but to a lesser extent (Fig. 3, Model 2). The association between the PRS-BMI and parental concern of overeating at wave 3 was also partly due to the interaction with z-WC at wave 1; however, the proportion of the mediated interaction was weaker than the proportion of the mediation effect (9%, *p* = 0.01 and 42%, *p* < 0.001, respectively).

The overall proportion mediated of the association between the PRS-BMI and parental concern of overeating at wave 3 (Mediation Model 1) was 44% (*p* < 0.001) and 51% (*p* < 0.001) through z-BMI and z-WC at wave 1, respectively. Lower mediation percentages were observed in Mediation Model 2, when parental concern of overeating at wave 1 mediated the association between PRS-BMI and both obesity indices at wave 3 (19%, *p* < 0.001 for z-BMI; 20%, *p* < 0.001 for z-WC). Sensitivity plots of the models are shown in Fig. S1 (Additional files).

## Discussion

In this longitudinal analysis involving European children, we showed that BMI and WC predicted parental concern of overeating to a greater extent than vice versa. Further, we demonstrated that BMI and WC partly mediated the prospective association between the latest PRS-BMI and parental concern of overeating. We also showed that parental concern of overeating partly mediated the prospective association between the PRS-BMI and obesity indices, extending previous cross-sectional findings of eating behaviors as potential behavioral mediators of the genetic susceptibility to obesity.

Our study suggests a bi-directional association between parental concern of overeating and obesity. In particular, obesity indices predicted subsequent parental concern of overeating three to four times stronger than parental concern of overeating predicted subsequent obesity indices. Our results are largely consistent with previous studies of children using CLPMs to assess the directionality between eating behaviors and BMI [43, 44]. However, they used emotional overeating, which refers to eating more than needed when experiencing negative emotions [45], whereas our overeating construct was not related to emotional overeating but rather inquired about parental concern about their children eating too much and putting their child on a diet to prevent them from becoming overweight. In these previous studies, emotional overeating was bi-directionally associated with BMI during childhood, and the pathway from BMI to later emotional overeating was stronger. Similarly, in a study of Finnish adults, BMI and WC predicted greater changes in restrained eating than the other way around [46]. Additionally, another study of children aged 3 to 15 months showed bi-directional associations between appetite traits and weight in early infancy, but the pathway from appetite to subsequent weight was stronger than from weight to appetite [15], suggesting that the temporal associations between eating behaviors and obesity measures may differ during early infancy from those found in later childhood and adulthood.

The molecular and behavioral mechanisms [47] underlying most of the genetic variants associated with childhood obesity are still unknown [48]. The obesity-susceptibility gene FTO, which was discovered by a genome-wide association study [49], has been suggested to be involved in both metabolic and appetite pathways [50–52]. To our knowledge, previous research has not yet examined the temporal association between parental concern of overeating and obesity indices considering genetic susceptibility using PRSs. Previous mediation studies on the genetic susceptibility to obesity and eating behaviors have shown that high appetite and lower satiety responsiveness (behaviors similar to overeating) mediate the association between a PRS-BMI and obesity indices in early infancy and childhood [18, 19], suggesting that genes involved in appetite traits [53] may lead to weight gain. Nevertheless, negative reports also exist; for example, a prospective study of Norwegian children did not observe any mediation through appetite traits [54]. A previous study of monozygotic twin pairs discordant for BMI observed a reduced gray matter volume in the heavier twin [55]. These structural differences were involved in valuation and reward processes. Further, a recent study showed that obesity susceptibility genes may influence eating addiction and reward behaviors due to their strong expression in the insula and substantia nigra brain regions [56]. Hence, it seems that genes could affect appetite and satiety through the central nervous system leading to obesity, which further may lead to changes in the central nervous system [57].

Obesity susceptibility genes are not only expressed in the brain [53], but also in the adipose tissue [58], suggesting that there may be pathways other than eating behaviors by which obesity susceptibility genes might exert their influence. A recent American study of adults suggested physical activity, conscientiousness, education and depression as potential pathways by which genetic susceptibility to obesity may lead to weight gain [20]. As a novel aspect, we found that obesity indices mediate the prospective association between the PRS-BMI and parental concern of overeating. In line with this, a recent study of British adolescents showed that the association between different PRS-BMI and disordered eating behaviors was partly mediated through BMI [21]. Therefore, future research should examine pathways other than eating behaviors in the susceptibility to obesity, both on a behavioral but also on a physiological level.

We have to acknowledge some limitations of our study. First, causal mediation analysis relies on the assumption of no unmeasured confounding [40]. Our sensitivity analyses showed that unmeasured confounding might still exist, although we adjusted for a wide range of different confounders. Second, we used the PRS derived from Khera et al. [7] based on BMI data of adults and we applied it to children. The PRS from Khera is age-dependent and shows minimal associations with birthweight, but it was strongly associated with a gradient in weight that emerged in early childhood when the children were 3.5 years old [7]. This is similar to our children’s baseline age, which was 4.4 years old. Further, the use of a PRS with 2.1 million SNPs provided a more comprehensive assessment of genetic susceptibility than other PRSs used in previous studies.

Third, children did not participate in all study waves, and dropout at follow up in our sample is associated with overweight, lower well-being scores or lower parental education which may have led to an underestimation of the associations between overeating and obesity indicators [59]. In addition, parents of overweight children tend to underestimate the weight status of their children [60]. Fourth, parental report of children’s eating behavior may be biased due to concerns about child’s weight or social desirability [61, 62]. It is not surprising that parents who are concerned about their child’s weight are more likely to adopt changes in their child’s feeding practices [62], which may explain why obesity indices predicted subsequent parental concern of overeating more strongly. Future research is needed to carefully check the temporal relationships between obesity indices and objective measures of overeating. Further, the eating behavior questionnaire used in this study aimed to assess parental feeding practices related to overweight during early childhood [29]. In a previous study using the same study population at waves 1 and 2 [63] and the same eating behavior questionnaire [29], the Cronbach’s alpha for the construct “parental concern for overweight and overeating” showed good internal consistency (Cronbach’s alpha = 0.82) [62]. Furthermore, in this previous study the construct “parental concern for overweight or overeating” was related to overweight and obesity longitudinally [63]. Fifth, this study followed a modular approach and some data was only available for subgroups [24], hence some examination modules had fewer study participants resulting in smaller sample sizes. Finally, our sample consisted of European children and replication of our findings in non-European populations is warranted.

The strengths of the present study include its prospective design, which allowed for the examination of the temporal relationship between parental concern of overeating and obesity indices. The inclusion of three time points in our CLPM analyses provided information on eating behaviors and obesity indices when the children were 4, 6 and 10 years old, respectively. Moreover, our analyses were not limited to a single country as we included children from eight European countries.

In conclusion, the findings of this study suggest that the associations between parental concern of overeating and obesity indices are bi-directional, but the pathway from obesity to parental concern of overeating is about three- to four-fold stronger than vice versa. By bridging nutritional and genetic research, this study increases our understanding of the temporal relationships in children’s genetic susceptibility to overeating and obesity. Future prospective studies are needed to replicate and explain these findings in larger samples of culturally and genetically diverse populations of children.

## Supplementary Information

**Additional file 1: Table S1.** Eating behavior patterns and factor loadings in varimax-rotated principal components. **Table S2.** Baseline characteristics of the study sample of the three study waves (*n* = 1848). **Figure S1.** Sensitivity plots from causal mediation analyses.

## Data Availability

Data described in this study will be made available upon request from the corresponding author.

## Abbreviations

BMI: Body Mass Index
CFI: Comparative Fit Index
CIs: Confidence Intervals
CLPM: Cross-lagged Path Model
ISCED: International Standard Classification of Education
PRS: Polygenic Risk Score
RMSEA: Root Mean Square Error of Approximation
SD: Standard Deviation
SNP: Single Nucleotide Polymorphism
TLI: Tucker-Lewis Index
WC: Waist Circumference
z-BMI: Body Mass Index z-score
z-WC: Waist Circumference z-score
