# Supplementary material for: The temporal relationship between parental concern of overeating and childhood obesity considering genetic susceptibility: longitudinal results from the IDEFICS/I.Family study
Source: Int J Behav Nutr Phys Act. 2021 Nov 4;18:139. doi: 10.1186/s12966-021-01205-9 (PMC8567680; doi:10.1186/s12966-021-01205-9)
Supplement: Supplementary file 1 — Additional file 1: Table S1. Eating behavior patterns and factor loadings in varimax-rotated principal components. Table S2. Baseline characteristics of the study sample of the three study waves (n = 1848). Figure S1. Sensitivity plots from causal mediation analyses. [file 12966_2021_1205_MOESM1_ESM.docx]

**Table S1.** Eating behavior patterns and factor loadings in varimax-rotated principal components.

|  | **Wave 1 (n = 13,219)** | | | | **Wave 2 (n = 11,061)** | | | | **Wave 3 (n = 4,805)** | | | |
| --- | --- | --- | --- | --- | --- | --- | --- | --- | --- | --- | --- | --- |
|  | **Parental concern of overeating** | **Low Appetite** | **Structure during feeding practices** | **Pushing the child to eat more** | **Parental concern of overeating** | **Low Appetite** | **Structure during feeding practices** | **Pushing the child to eat more** | **Parental concern of overeating** | **Low Appetite** | **Structure during feeding practices** | **Pushing the child to eat more** |
| Eigenvalue | 3.56 | 2.70 | 1.38 | 1.01 | 3.63 | 2.69 | 1.41 | 0.98 | 3.69 | 2.41 | 1.17 | 0.88 |
| Variance explained | 0.27 | 0.21 | 0.11 | 0.08 | 0.28 | 0.21 | 0.11 | 0.08 | 0.31 | 0.20 | 0.10 | 0.07 |
| **Variables** |  |  |  |  |  |  |  |  |  |  |  |  |
| How often does your child eat while doing something else? | -0.04 | -0.06 | **0.66** | 0.05 | -0.03 | -0.05 | **0.70** | 0.01 | 0.00 | -0.08 | 0.03 | **0.79** |
| Child watches TV at meals | 0.01 | 0.01 | **0.66** | -0.03 | 0.01 | 0.01 | **0.67** | -0.07 |  |  |  |  |
| Is it a struggle to get your child to eat? | 0.00 | **0.55** | -0.01 | 0.00 | -0.01 | **0.52** | -0.02 | -0.04 | 0.00 | **0.55** | -0.01 | -0.02 |
| Do you feed your child yourself if he/she does not eat enough? | -0.02 | 0.20 | 0.09 | **0.41** | 0.03 | 0.23 | 0.12 | **0.38** | -0.01 | 0.12 | **0.46** | 0.22 |
| Do you have to stop your child from eating too much? | **0.51** | -0.05 | -0.03 | -0.02 | **0.49** | -0.06 | -0.02 | -0.05 | **0.47** | -0.06 | 0.00 | -0.07 |
| Do you think about putting your child on a diet? | **0.52** | 0.04 | -0.03 | -0.09 | **0.50** | 0.01 | -0.03 | -0.09 | **0.50** | 0.02 | -0.08 | -0.01 |
| Do you make your child eat all the food on his/her plate? | -0.03 | -0.06 | 0.07 | **0.62** | 0.01 | 0.09 | 0.06 | **0.54** | -0.06 | -0.01 | **0.60** | 0.12 |
| Do you worry that your child is eating too much? | **0.54** | -0.01 | -0.01 | -0.01 | **0.52** | -0.03 | -0.02 | -0.04 | **0.52** | -0.02 | -0.01 | -0.03 |
| Do you worry that your child is not eating enough? | 0.00 | **0.51** | 0.01 | 0.06 | -0.01 | **0.50** | -0.01 | 0.01 | -0.02 | **0.52** | 0.02 | -0.01 |
| Do you use foods that your child likes as a way to get your child to eat “healthy” foods? | 0.11 | 0.13 | 0.08 | **0.40** | 0.17 | 0.23 | 0.10 | **0.33** | 0.18 | 0.21 | 0.28 | 0.22 |
| Does your child have a poor appetite? | -0.01 | **0.59** | -0.06 | -0.08 | -0.04 | **0.55** | -0.09 | -0.12 | 0.00 | **0.59** | -0.06 | -0.10 |
| Do you sit down together with your child when he/she eats meals? | -0.06 | -0.13 | **-0.32** | **0.47** | -0.08 | -0.21 | -0.14 | **0.64** | 0.03 | -0.12 | **0.58** | **-0.48** |
| Does your child eat too much when you are not around him/her? | **0.39** | -0.01 | 0.06 | 0.19 | **0.44** | 0.01 | 0.03 | 0.14 | **0.46** | 0.03 | 0.05 | 0.09 |

Eating behaviors with factor loadings (≥0.30) are in bold.

**Table S2.** Baseline characteristics of the study sample of the three study waves (n = 1,848)

|  | **Wave 1** | **Wave 2** | **Wave 3** |
| --- | --- | --- | --- |
| Age in years, mean (SD) | 4.4 (1.1) | 6.3 (1.1) | 10.1 (1.0) |
| Girls, n (%) | 884 (47.8) | 884 (47.8) | 884 (47.8) |
| Country, n (%)  Belgium  Cyprus  Estonia  Germany  Hungary  Italy  Spain  Sweden | 124 (6.7)  123 (6.7)  315 (17.1)  253 (13.7)  159 (8.6)  305 (16.5)  216 (11.7)  353 (19.1) | 124 (6.7)  123 (6.7)  315 (17.1)  253 (13.7)  159 (8.6)  305 (16.5)  216 (11.7)  353 (19.1) | 124 (6.7)  123 (6.7)  315 (17.1)  253 (13.7)  159 (8.6)  305 (16.5)  216 (11.7)  353 (19.1) |
| z-score BMI, mean (SD) | -0.01 (1.1) | 0.08 (1.2) | 0.36 (1.2) |
| z-score WC (SD)  Missing | -0.06 (1.2)  250 | 0.29 (1.3)  52 | 0.65 (1.2)  88 |
| Parental education level, n (%)  Low  Medium  High  Missing | 68 (3.6)  738 (39.2)  1028 (54.6)  50 (2.7) | 65 (3.5)  694 (36.8)  1068 (56.7)  52 (2.8) | 66 (3.6)  725 (39.2)  1057 (57.2) |
| Parental income level, n (%)  Low  Low - Medium  Medium  Medium - High  High  Missing | 258 (13.7)  283 (15.0)  509 (27.0)  307 (16.3)  405 (21.5)  122 (6.5) | 230 (12.2)  264 (14.0)  438 (23.2)  308 (16.3)  510 (27.1)  134 (7.1) | 286 (17.1)  112 (6.7)  613 (36.7)  212 (12.7)  446 (26.7) |
| Well-being score, mean (SD)  Missing | 40.9 (4.1)  118 | 40.2 (4.5)  106 | 40.3 (4.5)  - |
| Screen time (hours/week), mean (SD)  Missing | 9.7 (6.1)  72 | 12.2 (7.0)  74 | 14.6 (8.0)  - |
| Playing outside (hours/day), mean (SD)  Missing | 2.3 (1.5)  227 | 2.3 (1.3)  233 | 1.6 (1.1)  443 |
| Fruits and vegetables consumption (portions/week), mean (SD)  Missing | 18.5 (10.8)  229 | 18.9 (10.9)  227 | 19.0 (11.4)  - |
| Glycated hemoglobin (%), mean (SD)  Missing | 4.6 (0.5)  723 | 5.1 (0.3)  734 | 5.0 (0.3)  - |

**Figure S1.** Sensitivity plots from causal mediation analyses

**Model 1** through z-BMI at wave 1 (n = 1,246)

**Model 1** through z-WC at wave 1 (n = 1,134)

**Model 2** for z-BMI at wave 3 (n = 2,386)

**Model 2** for z-WC at wave 3 (n = 1,983)
